# Effects of Treatment with Zofenopril in Men and Women with Acute Myocardial Infarction: Gender Analysis of the SMILE Program

**DOI:** 10.1371/journal.pone.0111558

**Published:** 2014-11-03

**Authors:** Flavia Franconi, Stefano Omboni, Ettore Ambrosioni, Giorgio Reggiardo, Ilaria Campesi, Claudio Borghi

**Affiliations:** 1 Laboratory of Gender Medicine, National Institute of Biostructures and Biosystems and Department of Biomedical Sciences, University of Sassari, Sassari, Italy; 2 Clinical Research Unit, Italian Institute of Telemedicine, Varese, Italy; 3 Unit of Internal Medicine, Policlinico S. Orsola, University of Bologna, Bologna, Italy; 4 Mediservice, Milano, Italy; University of Perugia, Italy

## Abstract

**Background:**

the SMILE studies proved the prognostic benefit of zofenopril vs. placebo or other ACE-inhibitors (ACEIs) in post-acute myocardial infarction (AMI). In this retrospective pooled analysis of these studies we assessed whether the zofenopril effect is influenced by gender.

**Methods:**

the four double-blind, randomized, parallel-group SMILE studies, compared the efficacy and safety of 6–48 week treatment with zofenopril 60 mg/day with that of placebo, lisinopril 10 mg/day or ramipril 10 mg/day in 3630 AMI patients. This pooled analysis compared treatment efficacy (1-year combined occurrence of death or hospitalization for CV causes) in 2733 men and 897 women.

**Results:**

women were older than men, had a higher prevalence of diabetes and of other major CV risk factors. The risk of a major CV event was significantly larger for women (23% vs. 17% men, p<0.001). Between-gender risk difference was more marked for people living in Southern (+54%) than in Northern Europe (+12%). In both genders zofenopril similarly reduced the 1-year risk of CV morbidity and mortality vs. placebo (−39% men, p = 0.0001; −40% women, p = 0.005). The risk reduction was more marked with zofenopril than with the other ACEIs, particularly in men (−27%, p = 0.012; women: −14%, p = 0.479). The drug safety profile was similar between genders in zofenopril-treated patients, while it was worse in women treated with other ACEIs.

**Conclusions:**

post-AMI women are at higher risk of CV complications than men, particularly when living in Mediterranean countries. Their response to ACE-inhibition varies according to the type of drug and is usually better in men.

## Introduction

In the past years, gender differences in the access to health care resources and therapies have been extensively discussed, but little attention has been put on the different action of cardiovascular (CV) drugs [1], [2]. Historically, very few women have been enrolled and few clinical gender-specific analyses have been conducted during the development of CV therapies [3]. A certain number of CV agents have been approved for use in men and in women, but effects were evidenced only or predominantly in one of the two genders: the male one. Just as example, the statins have been approved in primary prevention of CV diseases but the scientific evidence was reached only in men (WOSCOPS) [4]. The under enrolment in trials for CV diseases still persists [5]. Most CV medications present a sexual dimorphism in pharmacokinetic and pharmacodynamic properties [6]. In particular, the renin-angiotensin aldosterone system (RAAS) is sexual dimorph. The ACE/AngII/AT(1)R and ACE2/Ang(1–7)/MasR and AT(2)R pathways are enhanced in men and in women, respectively [7]. The RAAS system is regulated by sexual hormones. In particular, it has been suggested that estrogen increases angiotensinogen levels and decreases renin levels, the activity of angiotensin-converting enzyme (ACE), density of AT(1) receptor and aldosterone generation [8]. Additionally, estrogen increases AT(2) receptor and angiotensinogen [1]–[7] and natriuretic peptides [8]. The influences of androgens on RAAS are less known, but testosterone seems to increase renin levels and ACE activity [7].

Clinically, one meta-analysis shows that ACE-inhibitors are less effective in reducing mortality in women with symptomatic heart failure than in men, whereas ACE-inhibitors do not modify the survival in women with asymptomatic heart failure [9]. In women at high CV risk, ACE-inhibitors reduce CV events when used as secondary prevention [10]. However, an Australian study evidences a decrease in CV events in men but not in women [11]. Furthermore, cough and angioedema are more frequent in women than in men during treatment with ACE-inhibitors [12], [13]. Notably, men but not women with the XPNPEP2 C-2399A genotype, characterized by high plasma levels of aminopeptidase inactivated metabolites, are susceptible versus ACE-inhibitors [14]. Whereas, ACE-inhibitor-related cough seems to be associated in a sex specific manner with polymorphism of the bradykinin receptor 2. Saliently, the majority of women and men discontinue ACE-inhibitor therapy because of cough and hypotension, respectively [15].

In general, the previous observations evidence that there is still the need to understand and overcome the gender differences in CV medicine and this need is specifically valid for drugs that interfere with RAAS and that are a mainstay CV therapy [16]. Therefore, we analyzed, with a gender approach, studies performed with zofenopril, an ACE-inhibitor with a high potency, significant tissue selectivity and a long duration of action [17], [18] and with lisinopril and ramipril. In doing that, a retrospective pooled analysis of the four double-blind randomized, prospective SMILE Studies (Survival of Myocardial Infarction Long Term Evaluation) was performed separately assessing treatment effect on men and women [19]–[22]. Notably, the SMILE studies individually proved the prognostic benefit of zofenopril reducing the 1-year occurrence of major CV events versus placebo or versus ramipril and lisinopril in men and women with acute myocardial infarction (AMI) [19]–[22]. The four SMILE studies enrolled patients in almost all European Countries, with a high heterogeneity for gender attention and awareness according to World Economic Forum, being the Northern Countries in very high positions, while Mediterranean Countries have a low position [23]. Indeed between the Northern and the Southern European Countries there are enormous differences in dietary habits which could deeply affect the fitness of CV system. Therefore data were analyzed also considering the geographical residency of the patients.

## Methods

### Study design and population

Detailed description of the study design and of the inclusion and exclusion criteria for each of the four SMILE trials is reported in the original publications [19]–[22]. Briefly, the four double-blind, randomized, parallel-group SMILE studies, compared the efficacy and safety of zofenopril with that of placebo (SMILE-1 and 3) [19], [21], lisinopril (SMILE-2) [20] or ramipril (SMILE-4) [22] in European men and non-pregnant women with AMI. Patients included in the studies were those with i) an early AMI (<24 hours), not eligible for thrombolytic therapy because of late admission to the intensive care unit or with contraindication to systemic fibrinolysis (SMILE-1) [19], ii) a confirmed diagnosis of AMI and a prior thrombolytic treatment within 12 hours of the onset of clinical symptoms of AMI (SMILE-2) [20]; iii) a recent AMI (within 6±1 weeks) with preserved left ventricular ejection fraction (>40%), treated with a thrombolytic treatment and with ACE-inhibitors (SMILE-3) [21]; and iv) an early myocardial infarction (<24 hours), treated or not with thrombolysis, with primary percutaneous transluminal angioplasty or coronary artery by-pass graft, and with clinical and/or echocardiographic evidence of left ventricular dysfunction (SMILE-4) [22].

All studies were conducted in accordance with the Guidelines for Good Clinical Practice and the Declaration of Helsinki and were approved by the Institutional Review Board of the University of Bologna as well as by the local ethics committees when required (a list of centers may be found in the original study publications) (19–22). Written informed consent was obtained from each patient before enrollment. Trial registration numbers are not available for SMILE-1 Study (protocol no. 31,188-07), SMILE-2 Study (Protocol no. ZOF-07) and SMILE-3 Study, because the studies were performed in the nineties, when registration was not mandatorily required. SMILE-4 Study (protocol MEN/03/ZOF-CHF/001) was registered with EudraCT Number 2004-001150-88 (www.clinicaltrialsregister.eu) and with the Italian Ministry of Health Code: GUIDOTT_III_2004_001 (https://oss-sper-clin.agenziafarmaco.it).

### Treatments

In all studies, patients were randomly allocated to treatment with zofenopril or comparator (placebo, lisinopril or ramipril). No lead-in observational period was foreseen prior to randomization, except for the SMILE-4 Study. In this study, patients meeting eligibility criteria entered a 4-day open label phase prior to randomization, when zofenopril was administered to all patients according to the up-titration scheme described above. This choice was based on ethical and regulatory reasons, based on previous evidence of efficacy and safety of the early zofenopril treatment in patients with anterior AMI [22]. Randomized active drug treatment was given in addition to standard recommended therapy for AMI. The initial dose of zofenopril was 7.5 mg twice daily and was progressively doubled up to the final dose of 30 mg twice daily if systolic blood pressure remained >100 mmHg and if there were no signs or symptoms of hypotension. Zofenopril up-titration was done according to the following scheme: 7.5 mg twice daily on day 1 and 2, 15 mg twice daily on day 3 and 4, 30 mg twice daily on day 5. The doses of the active comparators were up-titrated as well: up to 10 mg once-daily for lisinopril and up to 5 mg twice-daily for ramipril. Randomized treatment was continued for 6 to 48 weeks and patients were seen at enrollment and every 1 to 6 months, depending on the Study. For all studies, duration of treatment and follow-up periods overlapped, the only exception being represented by the SMILE-1 Study. In this trial, on completion of the 6 week double-blind treatment period, the patients stopped taking the study medication but continued treatment with their other medications for additional 48 weeks, at which time vital status was blindly evaluated.

### Statistical analysis

The present is a meta-analysis of individual patient data, based on raw data from each of the four SMILE Studies. The objective was to evaluate the impact of gender on treatment efficacy in patients with AMI treated with zofenopril, placebo or other ACE-inhibitors.

Since all the four SMILE Studies provided information on fatal and non-fatal CV events, the primary study endpoint of this retrospective analysis was set to the 1-year combined occurrence of death or hospitalization for CV causes. The efficacy end-point derived from the four Studies was calculated after weighing for the number of subjects contributing from each study. The efficacy analysis was carried out on the intention-to-treat population, made up of all randomized patients treated with at least one dose of study medication and documenting at least once the measure of the primary efficacy assessment, even in case of protocol violation or premature withdrawal from the study. The safety analysis was applied to all randomized patients, by assessing the incidence of adverse events and changes in laboratory data or ECG during the study. The measure of safety used in this pooled analysis was the rate of drug related adverse events expressed as the number of drug related adverse events divided by the person-time-at-risk throughout the observation period.

The baseline characteristics and the distribution of variables in the study populations and subgroups were compared using a Chi-square test for categorical variables and a Student t-test for continuous variables.

This meta-analysis was conducted using a one-step approach, which evaluates the individual patient data from all studies in a single step, whilst accounting for clustering of patients within studies [24]. The standard pair wise direct comparison approach was followed in order to asses differences among treatments [25]. Relative risk reductions and 95% confidence intervals were calculated by a Cox proportional-hazard regression model in which treatment group, gender (males vs. females), country, age (<65 years vs. ≥65 years), BMI (<30 vs. ≥30 kg/m^2^), diabetes (yes vs. no) and presence (yes vs. no) of a major CV risk factor (previous angina, previous congestive heart failure, hypertension, hypercholesterolemia, peripheral artery disease, prior coronary artery bypass graft percutaneous coronary intervention) were included as a covariate. In order to account for the different duration of follow-up among the four studies, the relative risk of CV morbidity and mortality was assessed using a time-dependent Cox regression model. Survival curves were drawn using Kaplan-Meier estimates and they were compared using the log-rank statistics. A subgroup analysis by country of origin (Northern vs. Southern Europe) was also made.

All p values are 2-sided and the minimum level of statistical significance was set at p<0.05. Data are shown as mean ± SD or as mean and 95% confidence interval or as absolute (n) and relative (%) frequencies. All analyses were performed by SAS software version 9.1.3. (SAS Institute INC., Cary, NC, USA).

## Results

### Patient population

Overall, 3630 patients from the four SMILE Studies were included in the pooled analysis, of which 1808 (50%) randomized to zofenopril, 951 (26%) to placebo, 520 (14%) to lisinopril and 351 (10%) to ramipril. As expected in case of trials including patients with cardiovascular diseases [3], men were more prevalent than women (75% vs. 25%, p<0.001); however, the gender distribution was homogeneous within each treatment group (**Table 1**). Women were older than men (66±10 vs. 60±11 years, p<0.001), had a slightly, but significantly, higher BMI (27.1±4.5 vs. 26.8±3.5 kg/m^2^, p = 0.049), a higher prevalence of diabetes (38% vs. 32%, p = 0.004) and of other major CV risk factors (86% vs. 80%, p<0.001) (**Table 1**). In addition, some heterogeneity was observed for age, BMI, rate of diabetes and of major CV risk factors across the four treatment groups, irrespective of the gender (**Table 1**).

**Table 1:** Demographic and clinical characteristics of the study population summarized by type of treatment and according to the gender.

	Zofenopril	Placebo	Lisinopril	Ramipril		All	
	n = 1808	n = 951	n = 520	n = 351	p-value	n = 3630	p-value
**Gender (n, %)**							
Men (n, %)	1357 (75)	705 (74)	395 (76)	276 (79)	0.396	2733 (75)	<0.001
Women (n, %)	451 (25)	246 (26)	125 (24)	75 (21)	0.752	897 (25)	
**Age (years, means±SD)**							
Men	60±11	62±10	57±10	59±11	<0.001	60±11	<0.001
Women	66±10	68±9	63±10	65±10	<0.001	66±10	
**BMI (kg/m^2^, means±SD)**							
Men	27±4	26±3	27±4	27±4	<0.001	27±4	0.049
Women	27±4	26±3	27±4	27±4	<0.001	27±5	
**Diabetes (n, %)**							
Men	440 (32)	209 (30)	190 (48)	41 (15)	<0.001	880 (32)	0.004
Women	165 (37)	102 (42)	47 (38)	22 (29)	0.267	336 (38)	
**Other major CV** **risk factors (n, %)**							
Men	558 (79)	337 (85)	198 (72)	1098 (81)	<0.001	2191 (80)	<0.001
Women	211 (86)	100 (80)	65 (87)	395 (88)	0.195	771 (86)	

Data are shown as absolute (n) and relative (%) frequencies for categorical variables and as means (±SD) for continuous variables. P-values refer to the statistical significance of the difference across the four treatments or between the two genders.

BMI: Body Mass Index; CV: Cardiovascular; SD: Standard Deviation.

The men to women ratio was similar (p = 0.071) in populations living in Northern (men: 74% vs. women: 26%) and Southern Europe (76 vs. 24%). People residing in the South of Europe were younger (63±11 vs. 59±11 years, p<0.001), irrespective of the gender. They had a lower BMI in case of men (26.6±3.4 vs. 27.3±3.8 kg/m^2^, p<0.001), but higher in case of women (28.3±4.6 vs. 27.2±4.4 kg/m^2^, p<0.001). The prevalence of major CV risk factors was lower in southern Europe, regardless of the gender (80% vs. 85% Northern Europe, p<0.001).

### CV outcomes according to gender and geographic location

**Figure 1:**
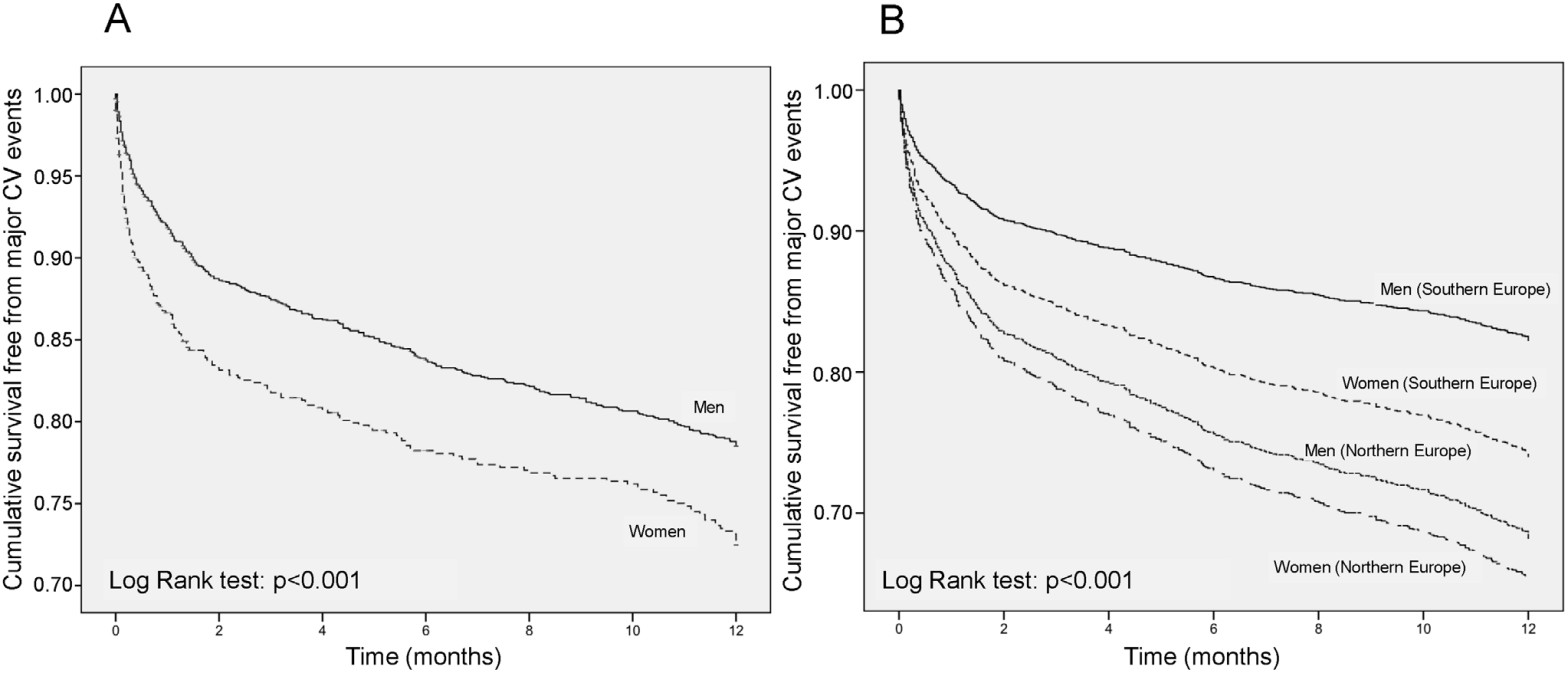
1-year survival rate according to gender. Kaplan-Meier cumulative survival curves during 1-year of follow-up in men and women enrolled in the SMILE Program. Data are shown for the whole study sample (A) and separately for individuals living in Northern and Southern Europe (B). CV: Cardiovascular.

During the 1-year follow-up, CV deaths or hospitalization occurred more frequently (p<0.001) in women 210/897 (23.4%) than in men 470/2733 (17.2%), with an 18% lower risk in the latter group [adjusted odds ratio and 95% confidence interval: 0.82 (0.69, 0.97), p = 0.021]. Consequently, men were more likely to survive than women during the observation period, the mean survival time being 10.2 months (95% confidence interval: 10.0, 10.3) vs. 9.6 months (9.3, 9.9) for women (p<0.001) (**Figure 1** **, panel A**). The risk of major cardiovascular outcomes was 28% [odds ratio: 0.72 (0.56, 0.93), p = 0.0139] larger in diabetic women [21%, mean survival time: 9.9 (9.4, 10.4) months] than in diabetic men [15%, mean survival time: 10.3 (9.9, 10.6) months].

Despite a higher quality of life in Northern Europe (Numbeo index 177.5 vs. 96.6 Southern Europe) [26], the incidence of major CV events was significantly larger in people leaving in the North (19.7%) than in those leaving in the South of the continent (18.2%) [odds ratio and 95% confidence interval: 1.80 (1.53, 2.11, p<0.001]. Notwithstanding the higher gender rating of the Northern European as compared to the Southern European countries [23], women were more prone to CV complications than men, regardless of the European country of origin. However, between-gender differences in the incidence of CV outcomes were more marked for people living in Southern [women 24.3% vs. men 16.3%; odds ratio and 95% confidence interval: 1.54 (1.25, 1.89), p<0.001] than in Northern Europe [22.1% vs. 18.8%; odds ratio and 95% confidence interval: 1.12 (0.86, 1.47), p = 391] (**Figure 1** **, panel B**).

### CV outcomes according to gender and treatment

**Figure 2:**
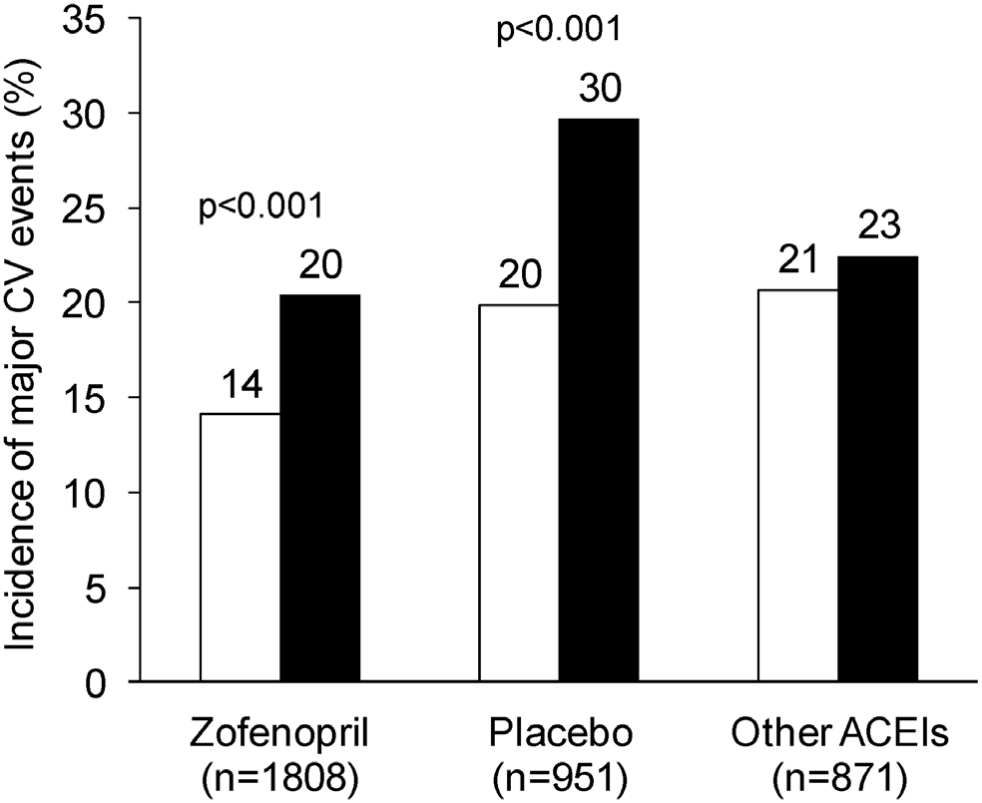
1-year incidence of cardiovascular events according to treatment and gender. Overall incidence (%) of major cardiovascular (CV) events during the 1-year follow-up in men (open bars) and women (full bars) treated with zofenopril, placebo or other angiotensin-converting-enzyme inhibitors (ACEIs). P-values refer to the statistical significance of between-gender difference.

The cumulative incidence of CV deaths or CV events was always larger in women than in men, irrespective of the type of treatment (**Figure 2**). This was particularly the case for subjects treated with placebo, for which 73 events were observed in women (29.7%) and 140 in men (19.9%) [odds ratio and 95% confidence interval: 1.70 (1.22, 2.37), p<0.001].

**Figure 3:**
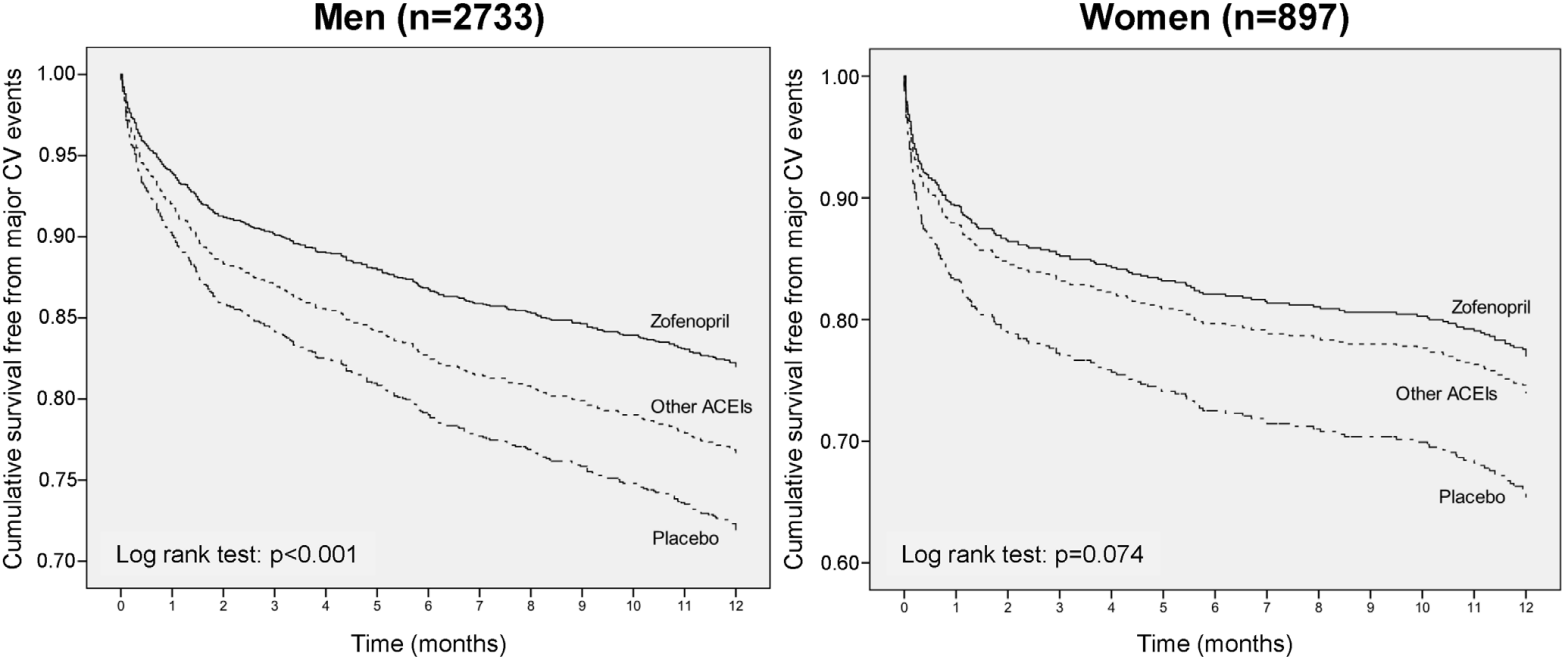
1-year survival rate according to treatment and gender. Kaplan-Meier cumulative survival curves during 1-year of follow-up in men and women enrolled in the SMILE Program and treated with zofenopril, other angiotensin-converting-enzyme inhibitors (ACEIs) or placebo. CV: Cardiovascular.

**Figure 4:**
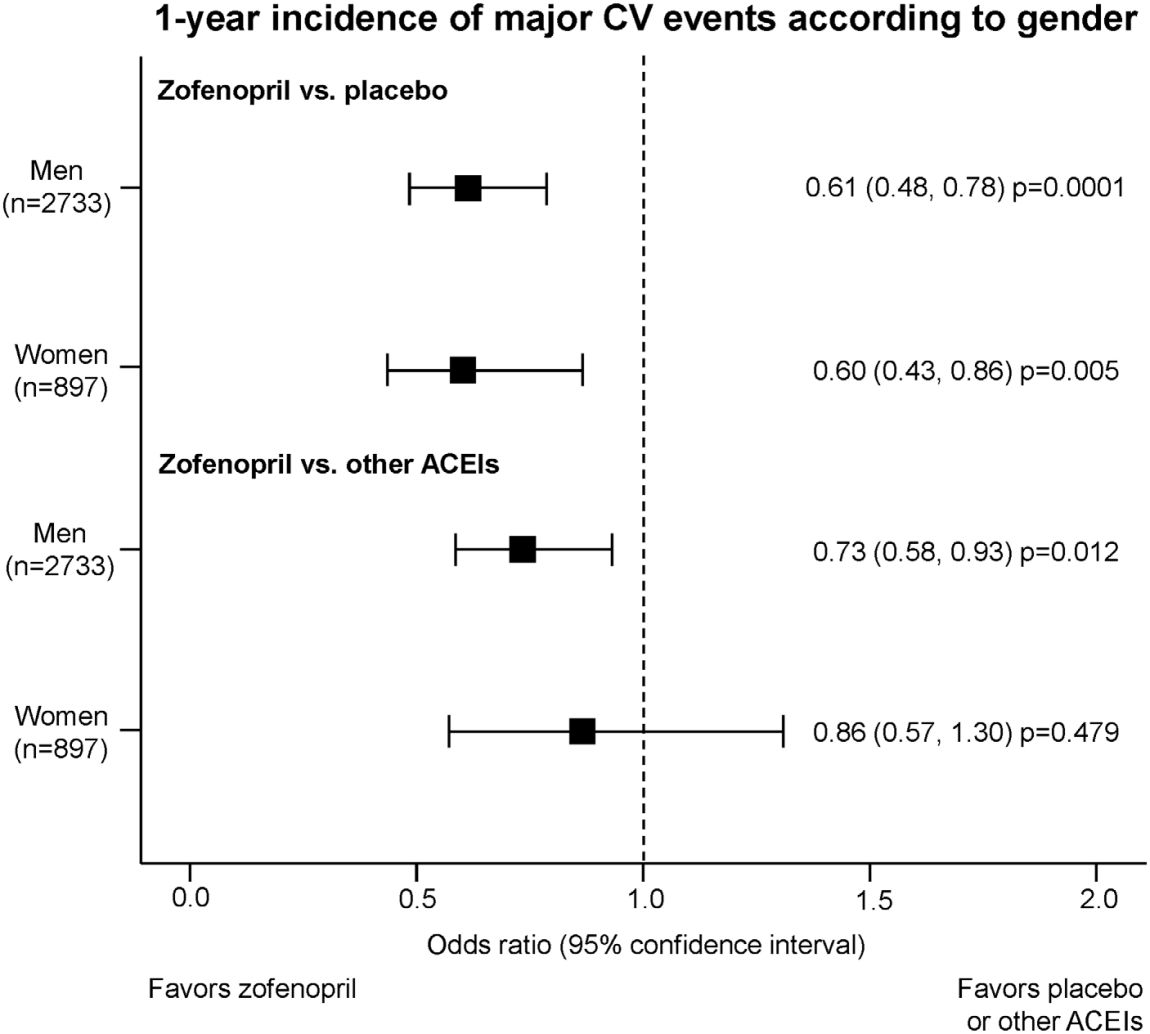
1-year risk of cardiovascular events according to treatment and gender. Effect of zofenopril vs. placebo or other angiotensin-converting-enzyme inhibitors (ACEIs) on the 1-year risk of major cardiovascular (CV) events according to gender in the SMILE Program. Data are reported as odds ratio and 95% confidence interval, with corresponding p value.

In either men or women zofenopril treatment was associated with a significantly larger reduction in the risk of 1-year CV morbidity and mortality and an improved survival as compared to placebo [odds ratio and 95% confidence interval for men: 0.61 (0.48, 0.78), p = 0.0001; women: 0.60 (0.43, 0.86), p = 0.005) (**Figure 3** and **4**). Between-gender difference in treatment effect vs. placebo lacked of statistical significance.

As compared to the other ACEIs, the reduction in the risk of major CV events with zofenopril was more marked for men [odds ratio and 95% confidence interval: 0.73 (0.58, 0.93), p = 0.012] than for women [0.86 (0.57, 1.30), p = 0.479]. Kaplan-Meier curves showed higher 1-year survival rates with zofenopril, with statistically significant differences being observed for men (**Figure 3** and **4**).

### Safety profile

Assessment of non-CV adverse events was done in 3697 patients (1841 treated with zofenopril, 956 with placebo, 520 with lisinopril and 380 with ramipril). Overall, a similar proportion of women (150/921, 16.3%) and men (449/2776, 16.2%) reported adverse events attributed to study treatment (p = 0.936 between genders), with some differences being however observed within each treatment group.

In placebo-treated patients, the incidence of adverse events was slightly, but not significantly larger in women than in men (13.0% vs. 9.9.%, p = 0.174). Under zofenopril, the rate of drug related adverse events, expressed by person-time at risk, was comparable in men (0.61) and women (0.56, p = 0.328), whereas in patients treated with other ACEIs it was higher in women than in men (0.67 vs. 0.50, p = 0.009). Cough, an adverse event which may typically be observed in ACE-inhibitor-treated patients, was reported in a similar low proportion by women and men treated with zofenopril and ramipril, while it occurred more frequently in lisinopril-treated women (7.2%) than men (2.8%, p = 0.025), indicating a possible intra-class difference for this type of event. The incidence of angioedema, was low and similarly distributed in women and men, regardless of the type of ACE-inhibitor taken into account (zofenopril: 0.4 vs. 0.7%; lisinopril: 0.8 vs. 0.8%; ramipril: 0 vs. 0.7%).

## Discussion

Although CV diseases have been considered a male disease for many years, actually they represent the leading cause of death also in women [27]. Indeed, there are numerous differences between men and women regarding diagnosis of CV diseases, and short- and long-term mortality rates. Women are also associated with poorer in-hospital and long-term prognosis [27]. In the present pooled individual analysis of four randomized, double-blind, prospective studies, including patients from several European countries, we confirmed that women with AMI are older than men and have more CV risk factors than men, including diabetes mellitus. Furthermore, we documented that the risk for CV hospitalization and death, is 18% higher, and survival free from events is lower, in women than in men. Our results confirm that in the modern era CV diseases are a natural killer also for women, and not exclusively for men [28].

Additionally, we demonstrated that living in the North of Europe increases the incidence of major CV events by 80%. The existence of a geographical North-to-South gradient in the prevalence and mortality of cardiovascular diseases in Europe is well-known and described in the literature [29]–[33]. The regional variations in cardiovascular diseases are caused by multiple factors such as differences between populations in CV risk factors as well as socio-economic factors, lifestyle variables such as diet, alcohol use, physical activity, medical care, genetic factors, and environmental conditions [34], [35]. The Current European Guidelines on Cardiovascular Disease Prevention in Clinical Practice take national variations in cardiovascular mortality into account [36].

Interestingly, the CV risk remains higher in women than in men, regardless of the geographical area, though the between-gender gap is higher in Southern than in Northern regions. This finding is in contrast with the myth that living in Mediterranean countries might be protective. Although we have no data to explain the origin of our finding we might speculate that women may have received less prompt and/or effective care than men, and this might be particularly the case for residents in Southern regions of Europe. Likely, the time has come for a major paradigm shift of ecological differences in health outcomes and for the fact that changes in the lifestyle and behavior might be essential in the causation of CV diseases [36]. Indeed, these results indicate that geographical localization should be considered in studies focused on gender aspects.

Undoubtedly, women remain underrepresented in most clinical trials and few clinical gender-specific analyses have been conducted during the development of CV therapies [3]. In this context, the retrospective evaluation of the SMILE studies according to gender confirms and expanded the current knowledge on gender differences in drug response. We confirmed that the proportion of women enrolled into the study, although in line with that of many other studies, is still poorly represented [37], [38]. We also showed that in post-AMI patients, the effect of ACE-inhibition in terms of CV outcome prevention was better in men than in women, supporting findings from previous studies in patients with CV disease [9], [11].

Additionally, our study provided new interesting findings. The gender differences in the response to treatment seem to depend on the geographical location rather than on the pharmacological treatment itself, since such differences are smaller among people living in Northern Europe and larger in people residing in Southern Europe. The reduction in the risk of 1-year CV morbidity and mortality induced by zofenopril versus placebo was not associated with gender. However, when the effect of zofenopril was compared with that of the other ACE-inhibitors, it was larger in men than in women. Although gender-related differences in the response to treatment with the various ACE-inhibitors used in our study might be related to chemical and pharmacological features of the compounds and their interaction with gender-specific characteristics, we have no data to support any hypothesis on the origin of our findings. However, to our knowledge, such an intra-class difference in the CV effect of ACE-inhibitors has never been reported. Further studies should explore this aspect in the future.

Cough and angioedema, two adverse drug reactions typically associated with ACE-inhibition and two of the major causes of withdrawal from ACE-inhibitor trials, are usually more frequently observed in women than in men [12], [13]. In our study, the incidence of cough, was similar between men and women treated with zofenopril, and ramipril, while it occurred more frequently in lisinopril-treated women, suggesting an intra-class difference. As far as zofenopril is regarded this confirms results of a previous pooled analysis in hypertensive patients, in which significantly more women than men experienced cough [39]. In the same meta-analysis, a slightly lower occurrence of cough was reported under zofenopril as compared to enalapril or lisinopril [39]. The incidence of angioedema, was low and similarly distributed in women and men, regardless of the type of ACE-inhibitor taken into account, suggesting no intra-class effect for this adverse drug reaction.

Our study has some limitations. First, although the design of the four SMILE studies was very similar, there were some differences in the inclusion criteria and treatment duration and follow-up which might have biased the study results, particularly when direct comparisons between different active drug treatments were made. Additionally, the primary endpoint used in this pooled analysis was not homogeneously shared among the four SMILE studies. The SMILE 1 and 4 studies primarily assessed the occurrence of death or major cardiovascular outcomes, while the SMILE-2 and 3 study evaluated the occurrence of severe hypotension and of cardiac ischemia, myocardial infarction or need for revascularization procedure, respectively, though in such studies death and/or hospitalization were assessed as secondary outcomes. We attempted to adjust such differences by standardizing the different study endpoints in one homogeneous outcome and by taking into account possible confounding variables such as age, gender, major CV risk factors, duration of follow-up and study sample; we also used individual patients’ data instead of averages. Second, we must acknowledge that some gender differences in response to ACE-inhibition might be explained by an imbalance in the number of subjects among the various treatment groups. For instance, the fact that the risk reduction observed with zofenopril in comparison with other ACE-inhibitors was significant in men, but not in women, might be related to the fact that the number of zofenopril-treated individuals was larger than that treated with lisinopril or ramipril.

## Conclusions

The results of the pooled data analysis of the SMILE studies confirm that post-AMI women are at higher risk of CV complications than men, particularly when living in Mediterranean countries. Their response to ACE-inhibition varies according to the type of drug employed. The CV drug effects are usually better in men than in women.
